# Trends in primary brain tumour incidence and mortality in Ireland 1995–2019

**DOI:** 10.1007/s11845-026-04326-x

**Published:** 2026-04-07

**Authors:** Kathleen Bennett, Bernadine O’Donovan, Stephen Macnally, Paul Carroll, Niamh Kavanagh, Ailish Malone, Frances Horgan

**Affiliations:** 1 School of Population Health, RCSI University of Medicine and Health Sciences, Beaux Lane House, Lower Mercer Street, Dublin, D02 DH60 Ireland; 2 Neuro-Oncology, Beaumont Hospital, Dublin, Ireland; 3 Rehabilitation Medicine, National Rehabilitation Hospital, St Vincent’s University Hospital and the Royal Hospital Donnybrook, Dublin, Ireland; 4 Neuro-Oncology, Beaumont Hospital, RCSI Smurfit Building, Dublin, Ireland; 5 School of Physiotherapy, RCSI University of Medicine and Health Sciences, Dublin, Ireland

**Keywords:** Brain tumour, Cancer registry, Epidemiology, Incidence, Survival

## Abstract

**Background:**

Primary brain tumours are a source of mortality and morbidity which require extensive resources, diagnostic and therapeutic technology. There is evidence that people with brain tumours can benefit from rehabilitation, but frequently experience difficulty accessing such service. The aim of this study is to examine incidence, patient and tumour characteristics and survival in adult primary brain tumours by major subtypes in Ireland.

**Methods:**

Data on all adult Irish patients diagnosed with primary brain tumours between 1995 and 2019 were extracted from the National Cancer Registry Ireland (NCRI). Descriptive statistics are presented by sub-type of brain tumour. Incidence rates were calculated by year period of diagnosis. Survival analysis was conducted using Cox proportional hazards regression.

**Results:**

Between 1995 and 2019, a total of 13,122 primary brain tumour cases in those aged 15+ years, were registered in Ireland. There were increases in diagnosed brain tumours over the 25-year period; 1,799 cases in 1995–1999 increasing to 3,590 in 2015–2019. Glioblastoma (WHO grade IV) was the most frequent tumour subtype (*n* = 3,671, 28%), followed by meningiomas (3,281, 25%), and other astrocytic tumours (1391, 10.6%). Overall survival rates increased over time, however, increasing age was associated with decreased survival. Survival was improved in females and those receiving surgery, radiotherapy or chemotherapy.

**Conclusion:**

The increased incidence and improved survival in major subtypes of brain tumours in Ireland over time is likely due to a variety of factors such as improvements in diagnostics, treatment advances and cancer registrations.

**Supplementary Information:**

The online version contains supplementary material available at 10.1007/s11845-026-04326-x.

## Highlights

Age-standardised rate (ASR) of incidence of brain tumours increased from 1995 to 2019.

Improved survival over time and in females compared to males.

Decreased survival with increasing age at diagnosis.

**Supplementary Information:**

The online version contains supplementary material available at 10.1007/s11845-026-04326-x.

## Introduction

 Primary brain tumours are a complex group of conditions, which vary in tumour type, location, progression and impact on the individual. Incidence and prognosis of brain tumours are influenced by key factors such as morphological subtype, gender and age [1].

The majority of primary brain and central nervous system (CNS) tumours occur in the brain and the remainder in the meninges, spinal cord, intracranial endocrine glands, and other parts of the CNS [2]. Categorisation of tumours has evolved over time, however, clarity and improved understanding of definitions and terminology remain an issue for patients and the public. The World Health Organisation (WHO, 2021) Classification of Tumors of the Central Nervous System (CNS WHO) groups brain and spinal cord tumours into 13 major categories with multiple subtypes [3]. Primary malignant brain tumours account for over one third of all brain tumours and are linked to high rates of morbidity and mortality [4]. The Global Cancer Observatory (GLOBOCAN) 2020 estimates that brain and CNS tumours constitute 1.9% of all cancers and these tumours have been ranked 12th among the leading causes of cancer deaths (2.5% of all cancers) [5]. These tumours have a significant impact on health-care systems due to the attendant impairments – headaches, loss of vision, seizures, speech difficulties - and frequent loss of independent functioning [6–8]. Highly specialised multimodal care is generally required and early rehabilitation is important to engage neuroplasticity and reduce potential complications [9, 10].

Global studies have identified increasing trends in incidence and mortality from brain tumours in most high-income countries (Europe and America) in the last decades [2, 5–12]. Many low-income countries have limited diagnostic resources and data collection systems. However analysis of data from the Global Burden of Diseases, Injuries and Risk Factors (GBD) 2016 and 2019 studies indicated significant increases in brain tumour incidence in all WHO regions, with a 17% increase in incidence worldwide between 1990 and 2016 [2, 6]. This trend continued in 2019 with a significant rise in incidence rates in all regions − 347,992 new cases of brain tumour were recorded [6].

There was also evidence of the influence of age and socio-economic factors on incidence rates; with a higher incidence in older men and in high-income countries [2, 5–12]. The highest ASRs in both sexes were reported in Europe (7.9 per 100,000 in males vs. 5.6 per 100,000 in females) [6]. The incidence of major primary brain tumour types in England were reported from 1995 to 2017 [13]. The most frequent tumour subtype was glioblastoma (31.8%), followed by meningioma (27.3%). The age-standardized incidence for glioblastoma and meningioma increased from 1995 to 2013. Incidence rates of other astrocytic and unclassified brain tumours decreased from 1995 to 2007 and then stabilised [13].

Detailed epidemiology data have been widely reported in the United States and some European countries [10, 14]. It is likely that increased incidence in Europe is driven by advances in neuroimaging, the establishment of high quality population-based registries and a growing and ageing population where tumours can be common issues [12–14]. These increased case numbers and survival rates have significant implications for European healthcare systems [13, 14]. There is evidence that rehabilitation has a significant role in the care of people diagnosed with a brain tumour, improving function (motor and cognitive) and quality of life [15]. However there are difficulties within existing neuro-rehabilitation services for people diagnosed with a brain tumour, who often experience barriers accessing services [16].

Since 1994, the National Cancer Registry Ireland (NCRI) has recorded information on cancer incidence, treatment and survival in Ireland. The NCRI reports that 2% of all registered tumours were primary brain tumours [17, 18]. Epidemiological research is important as it can reveal changes in disease patterns and guide development of services to meet population need. However, there are few epidemiological studies describing incidence, characteristics and survival in adults with primary brain tumours in Ireland [19]. Given the therapeutic value of rehabilitation, there is a need to gather current epidemiological information to guide effective service provision for brain tumour survivors. This study aims to describe the characteristics, trends in incidence and survival for primary brain tumours in adults in Ireland from 1995 to 2019.

## Materials and methods

### Registry data

Data on all adults (aged 15 years and over) diagnosed with a primary brain tumour in Ireland between January 1st, 1995 and December 31st, 2019 were extracted from the National Cancer Registry in Ireland (NCRI). Years are grouped into 1995–1999, 2000–2004, 2005–2009, 2010–2014, 2015–2019 for the analysis due to small counts. Tumour registrations, based on pathological Tumour Nodes Metastasis (TNM), are collected from hospital medical records, by Electronic Cancer Data Registrars, who are based in hospitals throughout the country. All relevant patient, tumour and treatment details are entered to the NCRI Cancer Registration System (CRS) from a range of data sources. These include hospital histopathology reports, the Hospital Inpatient Enquiry system (HIPE), radiology and oncology departments, medical charts and death certificate data [20, 21].

For this study individuals were identified as diagnosed with a primary brain tumour using the International Classification of Diseases [version 10] (ICD-10) tumour sites C70, C71, D32, D33, D42, and D43. For those diagnosed with primary CNS lymphoma, ICD-10 code site was used along with the morphology codes for lymphoma. The NCRI data includes the main morphological subtypes, including benign and malignant tumours. Registration information that was obtained from either hospital (e.g. medical charts) or non-hospital (e.g. death certificates) sources was included.

Brain tumour morphological subtypes were based on the 2016 CNS WHO, and by most common types: glioblastoma, other astrocytoma (excluding glioblastoma), meningioma, oligodendroglioma, embryonal tumours, ependymal tumours, oligoastrocytoma, primary CNS lymphoma, malignant glioma, unclassified malignant, unclassified benign, unclassified uncertain, and other. The subtype “other” includes brain tumour morphologies that could not be assigned to any of the above. These brain tumour subtype groups were based on morphology and not tumour behaviour. Categorisation of the brain tumour subtypes is shown in Additional File 1: Table S1.

All tumours extracted from NCRI were analysed irrelevant of their pathological confirmation. Metastatic tumours were not included in the analysis. For treatment data, surgery, radiation therapy and medical oncology within 12 months of diagnosis or 1 month before diagnosis was reported. For surgery, this included tumour-directed excisional surgery or related destructive treatment within 12 months after (or 1 month before) diagnosis date. For any medical oncology treatment, this included chemotherapy, biological/targeted agents and steroids within 12 months after (or 1 month before) diagnosis date, and radiotherapy within 12 months after (or 1 month before) diagnosis date.

### Incidence rates

For each brain tumour subtype, the age-standardised incidence rates per 100,000 person-years were calculated to the European Standard Population (ESP 2013). The ESP are population weights that are commonly applied to compare incidence rates across European populations. Age-standardised incidence rates were calculated by year period (1995–1999, etc.) of diagnosis, for all brain tumours and the three most frequent subtypes of glioblastoma, meningioma and other astrocytic tumour. In addition, age-specific incidence rates were calculated in 5-year age groups in adults (aged 15 years or over) for all brain tumours and the same three subtypes, with separate lines representing year periods (1995–1999, etc.).

### Survival analysis

Kaplan-Meier (KM) survival curves were generated to assess differences in overall survival over time. KM curves for the year of diagnosis, age at diagnosis, sex, and morphological subtype were generated. Log-rank tests were performed to evaluate differences in survival curves. Follow-up for survival was censored at 31/12/2021 or at death, whichever occurred first, and five-year survival (proportion alive) is presented with 95% confidence intervals (CI) for specific comparisons. Multivariable Cox proportional hazards model assessing overall survival was performed and included the following patient and tumour characteristics: Year of diagnosis (earliest 1995–1999 reference), age at diagnosis (15–19 years reference), sex (M reference), morphological subtypes (glioblastoma reference) and treatment (surgery, radiotherapy and medical oncology – no treatment reference). Hazard ratios (HR) and associated 95% confidence intervals (CI) are reported. The Cox proportional hazard assumptions were tested using Schoenfeld residuals and were not found to be in violation. Significance at *p* < 0.05 is assumed for all analyses. Analysis was conducted using Stata Software (version 17).

###  Ethical approval

 NCRI has approval from the Health Service Executive to collect, classify, record, store and analyse information on all cancer patients under Statutory Order 19 of 1991. Separate ethical approval for this study was not required as data were provided anonymised.

## Results

### Patient and tumour characteristics

Between 1995 and 2019, a total of 13,122 primary brain tumour cases in those aged 15 + years, excluding spinal, endocrinal, and other CNS tumours, were registered in Ireland. There were 6,313 cases in men (48.1%) and 6,809 in women (51.9%) (see Table 1). The median age group at diagnosis was 60–64 years.

**Table 1:** Characteristics of individuals diagnosed with a primary brain tumour in Ireland 1995–2019

Characteristics	Groups	Number	%
Sex	Male	6313	48.11
	Female	6809	51.89
Age groups			
	15–24	584	4.45%
	25–34	890	6.78%
	35–44	1330	10.14%
	45–54	2024	15.42%
	55–64	2693	20.52%
	65–74	2950	22.48%
	75–84	2014	15.35%
	85+	637	4.85%
Year of diagnosis	1995–1999	1799	13.71
	2000–2004	2145	16.35
	2005–2009	2478	18.88
	2010–2015	3110	23.70
	2015–2019	3590	27.36
Death before censor date (31/12/2021)	Yes	8461	64.48
Tumour-directed excisional surgery or related destructive treatment within 12 months after (or 1 month before) diagnosis date.	Yes	6162	47.0
Received any medical oncology treatment, including chemotherapy, biological/targeted agents and steroids within 12 months after (or 1 month before) diagnosis date.	Yes	4748	36.2
Radiotherapy within 12 months after (or 1 month before) diagnosis date	Yes	2598	19.8

The number of diagnosed brain tumours increased gradually over the 25-year period, from 1,799 cases in 1995–1999 to 3,590 in 2015–2019. Around a third of all patients were diagnosed with the most aggressive brain tumour subtype glioblastoma (WHO grade IV) (*n* = 3,671, 28%), followed by the least aggressive subtype meningiomas (3,281, 25%), and other astrocytic tumours (1391, 10.6%) (see Table 2).

**Table 2:** General tumour characteristics of 13,122 individuals diagnosed with a primary brain tumour

Variables	Groups	Number	%
Brain tumour Morphological subtype	Glioblastoma (GBM)	3671	27.98
	Meningioma	3281	25.00
	Other astrocytic tumours	1391	10.60
	Oligodendroglia	536	4.08
	Primary CNS lymphoma	392	2.99
	Ependymal tumours	206	1.57
	Oligoastrocytoma	157	1.20
	Embryonal tumours	63	0.48
	Malignant glioma	368	2.80
	Other brain tumours	1426	10.87
	Unclassified neoplasm, malignant	1368	10.43
	Unclassified neoplasm/tumour cells, benign	157	1.20
	Unclassified neoplasm/tumour cells, uncertain whether benign or malignant	106	0.81
Behaviour	Malignant	8038	61.26
	Benign	4214	32.11
	Uncertain	870	6.63
Tumour site	C70 meninges	230	1.75
	C71 brain	7415	56.51
	D32 benign meninges	3133	23.88
	D33 benign brain and other parts of CNS	1081	8.24
	D42 Uncertain or unknown of meninges	219	1.67
	D43 uncertain or unknown of brain and CNS	651	4.96
	Lymphoma	393	2.99
Grade	I	2727	20.78
	II	806	6.14
	III	1234	9.40
	IV	2846	21.69
	Unknown	5199	39.62
	B-cell/T-cell (lymphoma)	310	2.37
European network Basis of diagnosis	0 = Death certificate only	143	1.09
	1 = Clinical only	33	0.25
	2 = Clinical investigation, incl. imaging	3631	27.67
	4 = Specific tumour markers	0	0
	5 = Cytology	16	0.12
	7 = Histology of a primary tumour	9055	69.01
	9 = unknown	244	1.86

### Trends in incidence

Age-standardised incidence rates increased by 35.4% over time from 79.2 in 1995–1999 to 107.2 per 100,000 population in 2015–2019 (see Table 3). Increases were consistent across most tumour sub-types including glioblastoma (71.4%, Table 4) and meningioma (96.9% increase) (see Table 4). However, there was a decline in age-standardised incidence rates for other astrocytic tumours (−36.4%).

**Table 3:** Age-standardised (AS) incidence rate and 95% CI (combined all brain tumours males/females)

Years	AS incidence rate per 100,000 population (15 + years)	Lower 95% CI	Upper 95% CI
1995–1999	79.2	75.6	82.6
2000–2004	88.1	84.3	91.8
2005–2009	91.8	88.2	95.4
2010–2014	104.1	100.4	107.8
2015–2019	107.2	103.7	110.7

**Table 4:** Age-standardised (AS) incidence rate and 95% CI for three most frequent sub-types of brain tumour

Brain tumour subtype	Years	AS incidence rate per 100,000 (15 + years)	Lower 95% CI	Upper 95% CI
Glioblastoma	1995–1999	19.2	17.3	21.0
	2000–2004	24.2	22.3	26.2
	2005–2009	27.6	25.6	29.7
	2010–2014	30.9	28.9	32.9
	2015–2019	32.9	31.0	34.9
Meningioma	1995–1999	16.1	14.4	17.8
	2000–2004	18.1	16.4	19.8
	2005–2009	23.3	21.4	25.1
	2010–2014	30.7	28.7	32.8
	2015–2019	31.7	29.8	33.6
Other astrocytic	1995–1999	12.1	10.8	13.5
	2000–2004	9.4	8.3	10.5
	2005–2009	8.1	7.1	9.1
	2010–2014	6.9	6.0	7.7
	2015–2019	7.7	6.8	8.6

The unclassified subtypes, i.e., without a known morphology, including other malignant glioma and other brain tumours, represented 26.1% of all brain tumour diagnoses. The proportion of tumours recorded as having an ‘unknown’ grade has decreased over time from almost 72% in 1995–1999 to 20% in 2015–2019 (See Additional file 1: Table S2).

### Survival

**Fig. 1:**
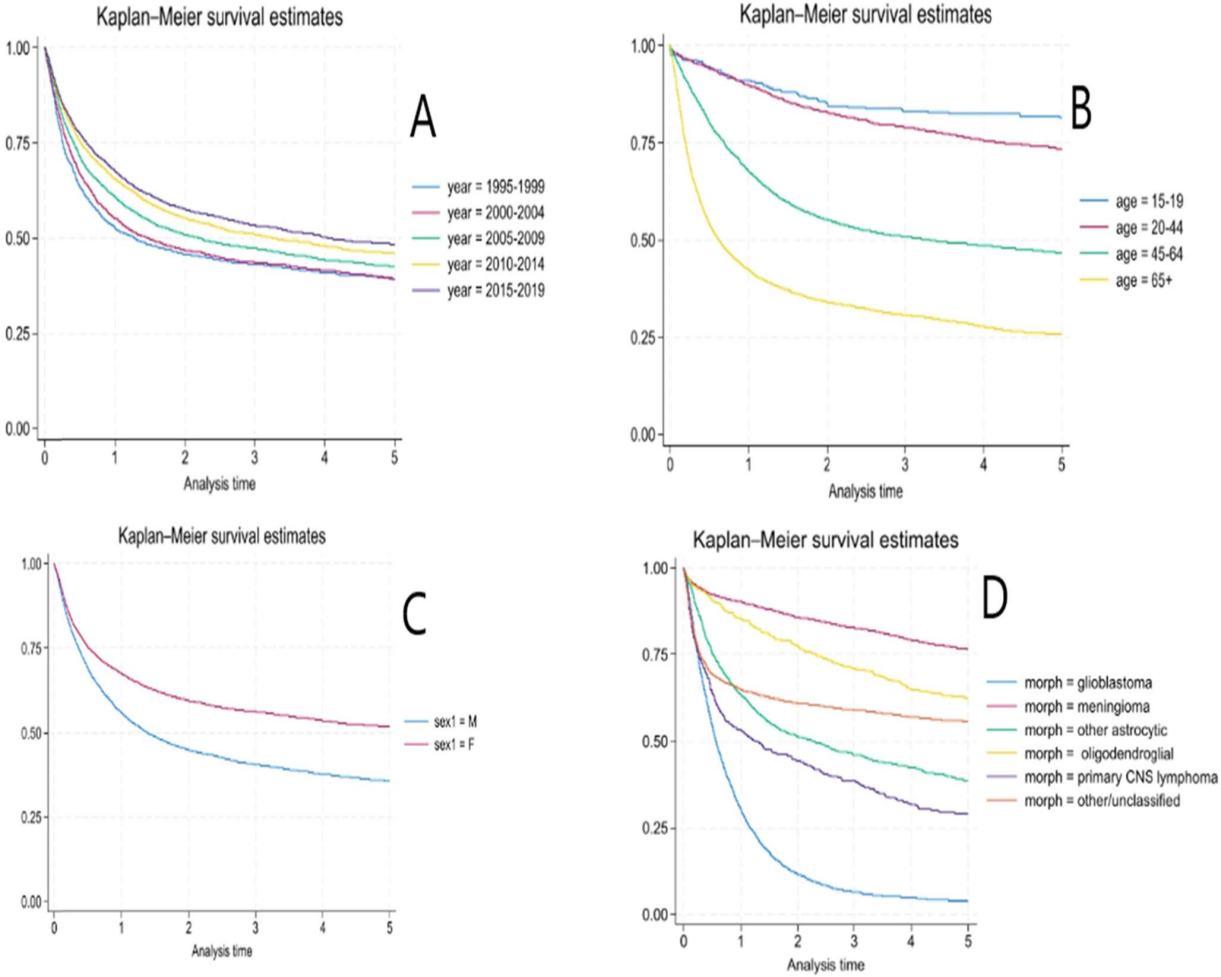
K-M plots (5 year survival) by year of diagnosis (**A**), age (**B**), sex (**C**) and brain tumour morphology (**D**)

Kaplan-Meier survival curves are shown in Fig. 1(A-D) for year of diagnosis (A), age at diagnosis (B), sex (C) and morphological subtypes (D). HR and 95% CI from a Cox proportional hazards regression model are shown in Table 5. There was a significant improvement in survival for primary brain tumour over time (trend adjusted HR = 0.91 (95% CI 0.89, 0.93) per period of years). Overall five-year survival increased from 39% (95% CI 37%, 41.5%) in 1995–1999 to 48% (95% CI 46%, 50%) in 2015–2019. Survival in females was significantly improved compared to males (adjHR = 0.87) with five-year survival in females 52% (95% CI 50%, 53%) compared to only 36% (95% CI 34%, 37%) in males. Survival decreased with increasing age at diagnosis, with five-year survival in those aged 20–44 years at 73.5% (95% CI 72%, 75%) compared to 26% (95% CI 25%, 27%) in the oldest age group. Most morphological tumour types had improved survival compared to glioblastoma, with the five-year survival only 3.7% (95% CI 3.1%, 4.2%). Those receiving surgery, radiotherapy or chemotherapy had improved survival outcomes compared to those that did not receive any interventions (see Table 5). Analysis of treatments indicated changes over time, with increased radiotherapy or chemotherapy from 1995 to 1999 to 2015–2019 (see Additional file 1: Table S3).

Survival curves for year of diagnosis (1995–2019) indicate improved survival over time (A). Among age groups (15–65+) there is decreased survival in the older age group (65+) (B). There is improved survival in females compared to males (C) and better survival in other tumour types compared to glioblastoma (D).

**Table 5:** Cox proportional hazards regression modelling of individual and tumour characteristics for overall survival (adjusted HR and 95% CI)

Characteristic		Adjusted HR (95% CI)	*p*-value
Year	1995–1999(ref) 2000–2004 2005–2009 2010–2014 2015–2019	1.0 1.04 (0.97, 1.12) 0.95 (0.88, 1.02) 0.87 (0.80, 0.94) 0.74 (0.68, 0.80)	0.28 0.18 0.001 < 0.001
Age-group	15–19 (ref) 20–44 45–64 65+	1.0 1.95 (1.52, 2.51) 4.58 (3.57, 5.88) 9.64 (7.50, 12.39)	< 0.001 < 0.001 < 0.001
Gender	M (ref) F	1.0 0.87 (0.83, 0.91)	< 0.001
Morphology	Glioblastoma (ref) Meningioma Other astrocytic Oligodendroglial Primary CNS lympoma Ependymal Oglioastrocytoma Embryonal Other malignant glioma Unclassified malignant Unclassified benign/UK Other brain	1.0 0.11 (0.10, 0.12) 0.65 (0.60, 0.71) 0.39 (0.34, 0.44) 0.53 (0.41, 0.68) 0.13 (0.10, 0.17) 0.35 (0.29, 0.43) 0.41 (0.28, 0.60) 0.60 (0.53, 0.69) 0.50 (0.45, 0.55) 0.13 (0.10, 0.16) 0.07 (0.06, 0.08)	< 0.001 < 0.001 < 0.001 < 0.001 < 0.001 < 0.001 < 0.001 < 0.001 < 0.001 < 0.001 < 0.001
Grade	I (ref) II III IV B/T-cell Unknown	1.0 1.15 (1.00, 1.32) 2.02 (1.81, 2.27) 2.04 (1.83, 2.27) 1.38 (1.04, 1.85) 1.68 (1.53, 1.84)	0.051 < 0.001 < 0.001 0.028 < 0.001
Any surgery	Yes vs. No	0.71 (0.67, 0.74)	< 0.001
Any medical oncology treatment	Yes vs. No	0.76 (0.71, 0.81)	< 0.001
Any radiotherapy treatment	Yes vs. No	0.84 (0.79, 0.89)	< 0.001

## Discussion

### Key Points

This study found an overall rise in the AS incidence rate of primary brain tumours diagnosed in Ireland between 1995 and 2019. Glioblastomas were the most frequent tumour subtype, followed by meningiomas. There was decreased AS incidence for other astrocytic and unclassified tumours, which may be attributed to increased precision in histological and molecular classifications over this time. This study also identified differences in overall survival rates; improved rates for females compared to males and decreased survival rates for older patients.

### Study findings – comparison and interpretation

Our study findings on incidence are similar to a recent paper which described the incidence of adult primary brain tumours in England [13]. Data from the National Cancer Registration and Analysis Service (NCRAS) was used to demonstrate increased incidence between 1995 and 2017. As with our study, glioblastoma and meningioma were the most common subtypes and the incidence of other astrocytic and unclassified brain tumours also declined over time [13].

Findings from our study are also comparable to epidemiological profiles in European and other countries which have shown similar increases [4, 10, 15]. Research with registry data from France (2000–2007) and four Nordic countries found increased incidence in primary tumours and gliomas in older patients in an earlier period from 1974 to 2003 [22, 23]. A multicentre study in Australia (2000–2008), which examined 13 pathology databases also found increased incidence for glioblastoma multiforme (GBM) and meningioma comparable to US and European data [24].

The overall increases in incidence over time are likely due to advances in clinical practice including improvements in healthcare access, diagnostic tools (such as imaging and molecular testing) and cancer registration [12–14]. The impact of other factors such as ageing populations, and higher socioeconomic status have also been linked to increased incidence [6, 25]. As with previous research [12, 23], our findings support the impact of specific tumour sub-types, namely glioblastomas and meningiomas, on increased incidence rates. Our findings of decreased incidence for other astrocytic tumours may be influenced by improved histo-molecular evaluation. This reduced incidence is likely a result of new molecular classifications which have upgraded astrocytic tumour [13, 24].

This study also identified a pattern of improved survival, which is similar to international trends of longer survival in European and Western Pacific regions than South-East Asian and African regions [6]. High and high-middle income regions experienced decreased global mortality and disability-adjusted life-year (DALY) rates, between 1990 and 2016 despite increased incidence rates [2, 4, 11, 24]. This pattern was also evident in 2019 data, with differences between high-income and low-income countries - longer survival in Western European and Pacific regions than African and South-East Asia regions [6, 7].

However, increased survival rates often lead to increased disease burden overall [4, 24]. In the global burden of disease study, patterns of increased disease burden were identified in 2016 with the highest burden in central Europe, tropical Latin America and eastern Europe [2]. Survival is multifaceted and improved survival can be linked to numerous factors, including increased access to care; improved diagnostics and treatments; changes in data collection; socio-economic differences; patient characteristics and tumour type. Cancer treatments have improved with advances in neuro-oncology surgery such as functional neuro-navigation systems, ultrasound surgery, intraoperative MRI scans and intraoperative cortical and subcortical mapping techniques [25]. Radiotherapy has also progressed with focused radiation modalities such as stereotactic radiotherapy (SRT) for recurrent brain tumour linked to improved survival [26]. The addition of tumour treating fields (TTFields) to cancer treatment for patients with glioblastoma has had significant survival benefits [27]. The incidence of certain tumour histologies rises with age and advanced age at diagnosis is a prominent predictor of worse survival [28, 29]. Significant gender differences in survival may be linked to differences in tumour types/grades - gliomas occur more commonly in males, while meningiomas are more common in females but occur at lower grades than males. In addition, susceptibility to risk factors (e.g. smoking, body weight), sex hormones or poorer responses to therapy differ between females and males [6, 30].

The National Cancer Control Program (NCCP) is responsible for regional cancer services in Ireland. Currently there are eight designated HSE cancer centres and over 30 voluntary Community Cancer Support Centres across the country. Brain tumour care is primarily provided in the National Neurosurgical Centre Beaumont Hospital in North Dublin, the North East of Ireland and nationally; and the Cancer Centre at Cork University Hospital across the West, Midlands and South of Ireland. These provide a range of services from multidisciplinary teams (MDT) and may include a neurosurgeon, radiation oncologist, medical oncologist, neurologist, neuropsychology, nurse specialist, speech and language therapist, dietician, social worker, occupational therapist, physiotherapist, pastoral care [31]. Rehabilitation can improve functional prognosis (motor and cognitive) but those with a brain tumour diagnosis often experience difficulties accessing specialist rehabilitation services [32, 33].

Our findings provide context for healthcare professionals and health decision makers to help prioritise and plan rehabilitation for people diagnosed with a brain tumour. The increasing incidence and improved survival suggests that growing numbers are living longer and surviving beyond their initial brain tumour diagnosis. Population level epidemiological data are informative, however there is also a requirement for individual level data to inform more personalised and tailored therapies to meet the needs of those with a brain tumour diagnosis.

However, our research has found evidence that prolonged and evolving rehabilitation needs are not being met with pronounced difficulties accessing key rehabilitation services in Ireland [33, 34]. These patterns of rehabilitation care are evident across healthcare systems. Retrospective analysis of a large cohort of Italian individuals diagnosed with a brain tumour showed a limited number received rehabilitation interventions during their first year post diagnosis [23].

The need for rehabilitation is further highlighted by the relatively young median age at diagnosis of people with a brain tumour. Over half are still of working age, and therefore require additional supports to return to work after diagnosis and treatment. There are significant benefits from rehabilitation at all levels of the ecological model, from individual to family to societal, to achieving return to work [16]. Previous studies in rehabilitation after acquired brain injury have demonstrated significant cost-benefit [35]. However, limited research has been conducted to date, on occupational needs of people diagnosed with a brain tumour who are not yet of retirement age [33]. Our recent review of rehabilitation needs and interventions, linked multimodal rehabilitation to improved function and health-related quality of life [33]. A variety of therapies including inpatient neuro-rehabilitation, community-based exercise and tailored cognitive interventions frequently had positive effects that persisted across physical and cognitive domains [33].

### Strengths and limitations

This study used NCRI registry data which represents the most complete and accurate source of data on cancer including brain tumours in Ireland and is used for benchmarking internationally. This robust data on brain tumourr cases, that span the last 3 decades, facilitated the identification of trends. However, the role of influential factors such as advances in treatments should be considered when comparing historical and current survival data. It should be noted that broad classification of data, for example, year periods and age groups limits more detailed interpretation of trends and factors associated with incidence and survival. Benign and uncertain brain tumours were included as this is relevant to clinical and rehabilitation burden and avoids potential bias. There were some issues with data as only primary diagnosis and treatment were reported. There was limited information on socio-economic risk factors and other characteristics. Data was also not available on significant effects such as disability and impact on education, work-life and relationships. This study focused on primary tumours and did not gather information on secondary disease (metastases) which is an important and evolving area. This is a noteworthy omission as secondary disease surpasses primary disease in frequency and improved treatments of secondary disease are emerging.

## Conclusion

Classification, treatment and epidemiology of need associated with brain tumours continues to evolve over time. These findings have wider relevance for neuro-oncology services in Ireland, which are continually responding to increasing incidence and survival. The information provided will assist with prioritising the supports required. It will also encourage healthcare providers and policy makers to adequately resource neuro-rehabilitation for people who have been diagnosed with a brain tumour. Such resources are essential for those who are living with functional difficulties in their survivorship journey.

## Supplementary Information

Below is the link to the electronic supplementary material.

Supplementary Material 1 (DOCX 28.0 KB)

## Data Availability

Data have been provided by the National Cancer Registry Ireland (NCRI).
