# Supplementary material for: Trends in primary brain tumour incidence and mortality in Ireland 1995–2019
Source: Ir J Med Sci. 2026 Apr 7;195(3):1267–76. doi: 10.1007/s11845-026-04326-x (PMC13342330; doi:10.1007/s11845-026-04326-x)
Supplement: Supplementary file 1 — Supplementary Material 1 (DOCX 28.0 KB) [file 11845_2026_4326_MOESM1_ESM.docx]

### Supplementary tables S1-S3

### S1: Categories of Brain Tumor subtypes according to tumor morphologies

| Brain Tumor subtypes | Morphology Codes |
| --- | --- |
| Glioblastoma (GBM) | 9440-9442, 9445 |
| Meningioma | 9530-9539 |
| Other astrocytic tumors (excluding GBM) | 9381, 9384, 9400-9411, 9420-9421, 9424, 9425 |
| Oligodendroglial tumors | 9450-9451 |
| Primary CNS lymphoma | 9590-9596, 9611-9728, 9735-9766, 9970-9971 |
| Ependymal tumors | 9383, 9391-9394 |
| Oligoastrocytoma | 9382 |
| Embryonal tumors | 9470-9477, 9490, 9500-9501, 9508 |
| Malignant glioma | 9380 |
| Unclassified neoplasm, malignant | 8000/3a |
| Unclassified neoplasm/tumor cells, benign | 8000/0a, 8001/0a |
| Unclassified neoplasm/tumor cells, uncertain whether benign or malignant | 8000/1a, 8001/1a |
| Other brain tumors | Uncategorized morphologies with recorded  site codes C70-71, D32-33, D42-43 |

^a^Behavior coded by /0 for benign tumors, /1 for unspecified, borderline, or uncertain behavior, and /3 for malignant tumors

(adapted from Wanis et al., 2021)

### S2: Grade by year of diagnosis

| Year | B-cell | T-cell | Grade I | Grade II | Grade III | Grade IV | Unknown | Total |
| --- | --- | --- | --- | --- | --- | --- | --- | --- |
| **1995-1999** | 17 (0.94%) | 3 (0.17%) | 126  (7.00%) | 47  (2.61%) | 95  (5.28%) | 218  (12.12%) | 1293  (71.87%) | 1799 |
| **2000-2004** | 39  (1.82%) | 0 | 141  (6.57%) | 72  (3.36%) | 144  (6.71%) | 368  (17.16%) | 1381  (64.38%) | 2145 |
| **2005-2009** | 57  (2.30%) | 6  (0.24%) | 413  (16.67%) | 127  (5.13%) | 391  (15.78%) | 417  (16.83%) | 1067  (43.06%) | 2478 |
| **2010-2014** | 93  (2.99%) | 2  (0.06%) | 940  (30.23%) | 214  (6.88%) | 359  (11.54%) | 748  (24.05%) | 754  (24.24%) | 3110 |
| **2015-2019** | 90  (2.51%) | 3  (0.08%) | 1017  (30.84%) | 346  (9.64%) | 245  (6.82%) | 1095  (30.50%) | 704  (19.61%) | 3590 |

### S3: Number and percentage of surgery, radiotherapy and medical oncology over time in primary brain tumour

| **Years** | **Surgery**  **N (%)** | **Radiotherapy**  **N (%)** | **Medical Oncology N(%)** |
| --- | --- | --- | --- |
| 1995-1999 | 635 (35.30%) | 489 (27.18%) | 146 (8.12%) |
| 2000-2004 | 1143 (53.29%) | 705 (32.87%) | 272 (12.68%) |
| 2005-2009 | 1324 (53.43%) | 930 (37.53%) | 480 (19.37%) |
| 2010-2014 | 1429 (45.95%) | 1161 (37.33%) | 731 (23.50%) |
| 2015-2019 | 1631 (45.43%) | 1463 (40.75%) | 969 (26.99%) |
| Chi-square test (statistic, p-value) | 179.00; p<0.001 | 109.52; p<0.001 | 367.25; p<0.001 |
